#  Pectus carinatum as the key to early diagnosis of Morquio A syndrome: a case report

**DOI:** 10.1186/s13256-021-02737-1

**Published:** 2021-04-05

**Authors:** Kento Yamauchi, Daishi Hirano, Miho Wada, Hiroyuki Ida

**Affiliations:** Department of Pediatrics, Jikei University School of Medicine, 3-25-8 Nishi-Shimbashi, Minato-ku, Tokyo, 105-8461 Japan

**Keywords:** Early diagnosis, Mucopolysaccharidoses, Pectus carinatum, Urine glycosaminoglycan

## Abstract

**Background:**

A 20-month-old Asian boy with normal growth presented with genu valgum, kyphosis, and pectus carinatum, with no neurological symptoms. No other symptoms suggestive of mucopolysaccharidoses, for example joint contracture and peculiar facies, were present.

**Case presentation:**

As part of our differential diagnosis we found elevated urine glycosaminoglycans, which triggered further investigation. Detailed examination showed flattening of the ribs, kyphoscoliosis and ovalization of the thoracolumbar vertebral body, strikingly short metacarpals, and very slight cardiac regurgitation. *N*-Acetylgalactosamine-6-sulfatase levels in the blood and dermal fibroblasts were very low, thus confirming diagnosis of Morquio A within 2 months of presentation. The patient was placed on elosulfase alfa enzyme replacement therapy and followed for 3 years.

**Conclusions:**

This case exemplifies the importance of considering mucopolysaccharidoses as part of the initial differential diagnosis of pediatric patients with skeletal deformities; urine glycosaminoglycan levels and a blood enzyme mucopolysaccharidoses panel are simple screening tests that could lead to early definitive diagnosis.

## Background

Morquio A syndrome, also called mucopolysaccharidosis IVA (MPS IVA; OMIM #253000), is a very rare autosomal recessive disorder characterized as an “inborn error of metabolism” [1]. Mutations in the *N*-acetylgalactosamine-6-sulfatase (GALNS) gene result in deficiency of this lysosomal storage enzyme; this then leads to failure to degrade the glycosaminoglycans (GAGs), namely keratan sulfate (KS) and chondroitin-6 sulfate (C6S), with subsequent accumulation, which then leads to various organ dysfunctions [2].

Morquio A syndrome has an unpredictable and heterogeneous age of onset, rate of progression, and type and severity of symptoms. The International Morquio A Registry reports that initial symptoms are recognized at a mean age of 2.1 years (standard deviation 1–3 years) [3]. Common presenting features include short stature, short trunk, kyphosis (“hunchback”), atlantoaxial dislocation, genu valgum (“knock-knee”), and pectus carinatum (“pigeon chest”), and at presentation there is generally no central nervous system impairment [4, 5]. As the patient ages there is often progressive and irreversible skeletal dysplasia [3], respiratory complications (sleep-disordered breathing, obstructive or restrictive lung disorder, which is particularly linked with mortality [6]), cardiovascular complications (stenosis and regurgitation associated with valve thickening), and hearing and vision loss [2, 4, 7]. Over time, this disease progression affects quality of life and survival prognosis. Therefore, early diagnosis and continual management of symptoms, including intervention with surgery at the appropriate time, are critical to patient well-being.

**Fig. 1:**
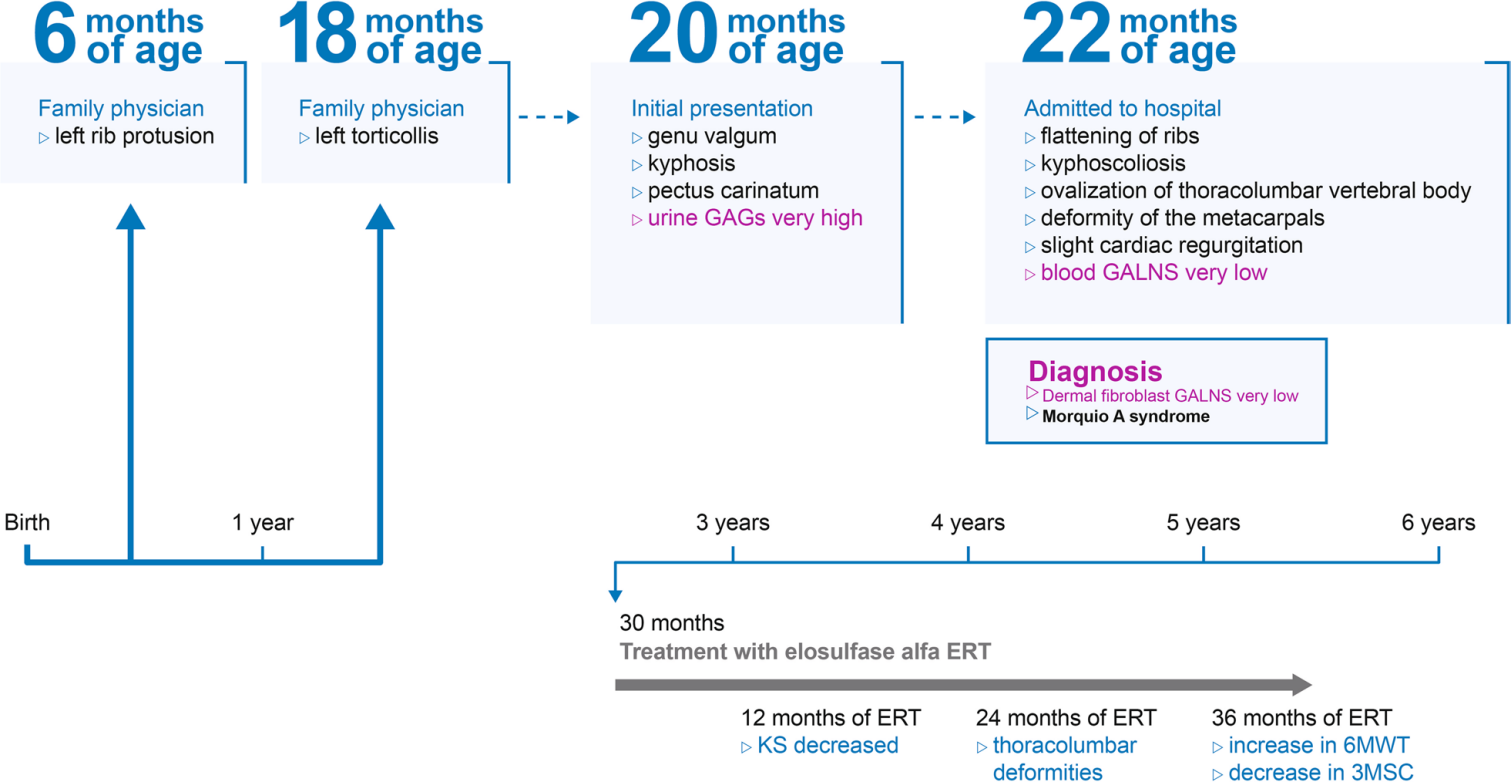
Overview of the case. 3MSC, 3-minute stair climb; 6MWT, 6-minute walk test; ERT, enzyme replacement therapy; GAGs, glycosaminoglycans; GALNS, *N*-acetylgalactosamine-6-sulfatase; KS, keratan sulfate.

However, at present it can take several years (mean 2.6 years [3]) from initial presentation to definitive diagnosis (mean age 4.7–4.9 years [3]), suggesting that disease management is inadequate. Here, we report a case of Morquio A syndrome in which definitive diagnosis was made within 2 months of initial presentation (an overview of the case is presented in Fig. 1).

## Case presentation

A 20-month-old Japanese boy presented with pectus carinatum with no relevant past or family medical history (including no history of metabolic/storage disorders) and as the second child in the family. He had presented to his family physician at 6 months of age with left rib protrusion, and at 18 months of age with left torticollis. At this time, chest X-ray showed deformity and protrusion of ribs (pectus carinatum), but there were no other symptoms suggestive of Morquio A syndrome; for example, joint contracture and peculiar facies were not present and there were no problems with sleep or respiration. The patient was referred to our department (Department of Pediatrics, Jikei University School of Medicine, Tokyo, Japan).

### Investigations

At the time of initial consultation in our department, the patient presented with normal growth for his age (height 83.3 cm, body weight 11.0 kg; 0.4 and 0.2 standard deviations, respectively, relative to growth standards for Japanese children [8]). On physical examination, we observed genu valgum, kyphosis, and pectus carinatum. Clear contracture of the elbow and knee joints was not observed. As part of our differential diagnosis based on these physical findings, and to rule out possible storage/metabolic diseases, we initiated a workup and measured urine GAG levels. We performed a quantitative urine mucopolysaccharide test and detected an elevation in GAGs: 97.7 µg/mL compared with a normal range of 5.3–34.0 µg/mL. The patient was admitted to the hospital for further examination.

**Fig. 2:**
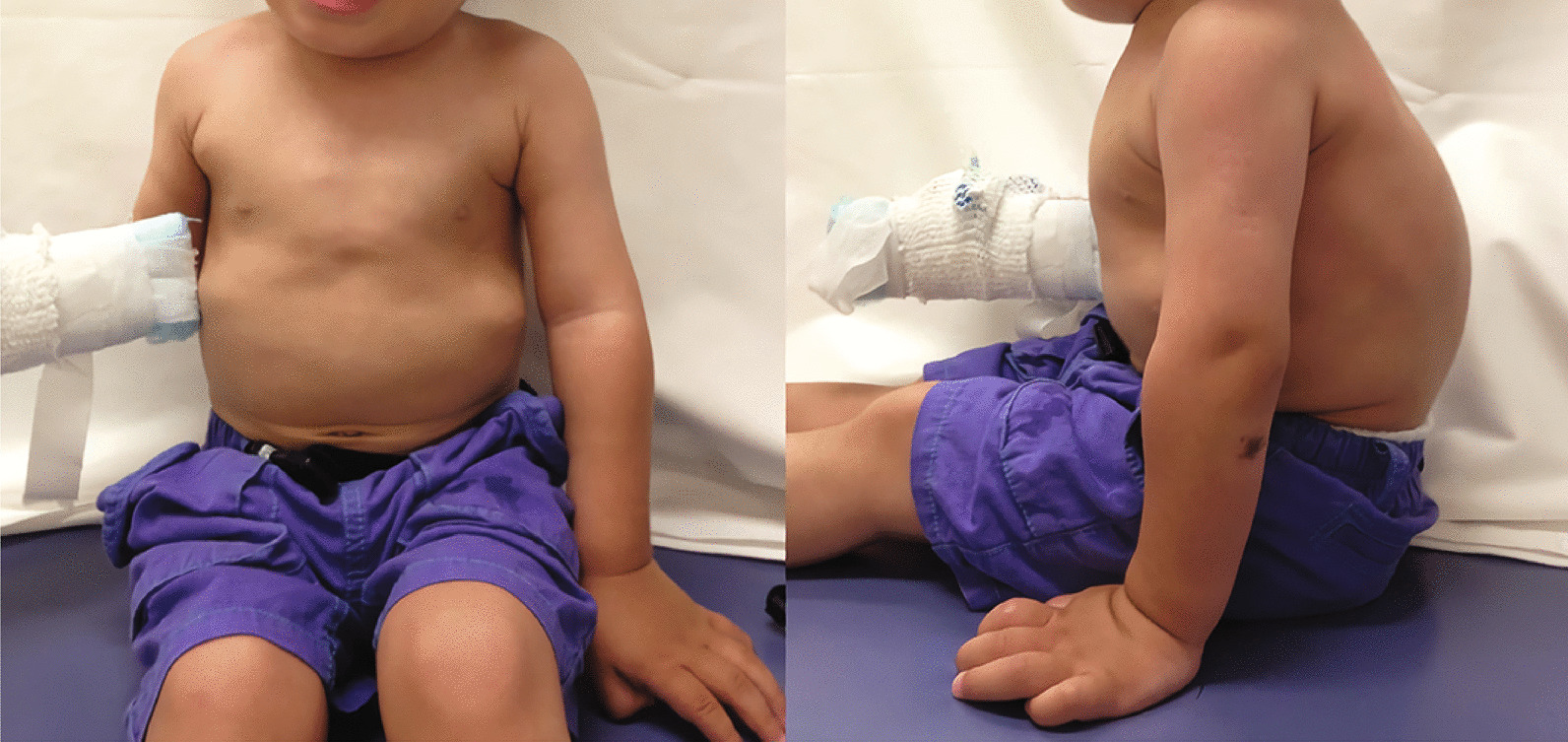
Physical features present at initial clinical examination. By the front view, the trunk is small for the head and extremities. By the lateral view, pectus carinatum, kyphosis, short neck, and large hands are evident.

**Fig. 3:**
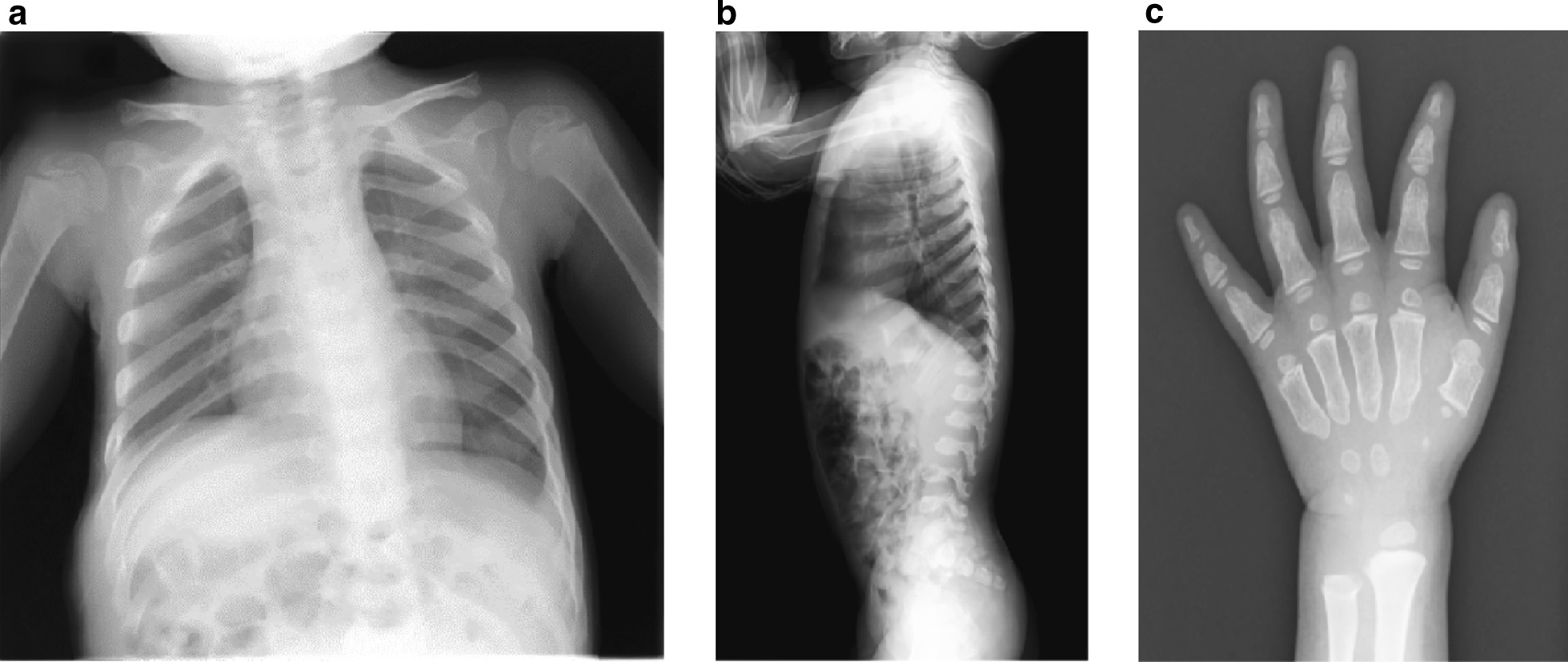
Skeletal features evident by **a** chest X-ray, **b** thoracolumbar X-ray, and **c** carpus X-ray. Chest X-ray showed the flattening of ribs (“oar-like” ribs). Thoracolumbar X-ray showed worsening of kyphosis and ovalization of the thoracolumbar vertebral body. Carpus X-ray showed short and stubby phalangeal bones with mild metaphyseal cupping, and strikingly short metacarpals with proximal coning.

On admission at 22 months of age, the patient presented with similar height and body weight and normal vital signs (body temperature 36.6 °C, blood pressure 94/- mmHg, heart rate 90 beats per minute, respiratory rate 35 breaths/minute). As at the initial consultation, genu valgum, kyphosis, and pectus carinatum were present (Fig. 2). Detailed clinical examination did not reveal adventitious lung sounds or heart murmur, hepatosplenomegaly, neurological abnormalities, coarse facial features, macroglossia, tonsillar hypertrophy, or umbilical hernia. Ophthalmic examination did not reveal any corneal opacity. Blood tests did not reveal any abnormalities. Chest X-ray showed the flattening of ribs (Fig. 3a; “oar-like” ribs), thoracolumbar X-ray showed kyphoscoliosis and ovalization of the thoracolumbar vertebral body (Fig. 3b), and carpus X-ray showed short and stubby phalangeal bones with mild metaphyseal cupping and strikingly short metacarpals with proximal coning (Fig. 3c). Echocardiography revealed very slight tricuspid, pulmonary, and mitral valve regurgitation. Brain magnetic resonance imaging (MRI) did not reveal any clear abnormalities in the parenchyma.

Given these findings, blood enzyme activity for MPS I, MPS II, MPS IVA, and MPS VI was measured (Advanced Clinical Research Centre & Asian LSD Centre, Institute of Neurological Disorders, Tokyo, Japan). GALNS enzyme activity, indicative of MPS IVA, was found to be at very low levels (0.05 pmol/punch/hour) compared with normal levels (1.21–1.87 pmol/punch/hour). The enzyme activity levels for MPS I, MPS II, and MPS VI were within the normal range.

### Differential diagnosis

Given the low levels of GALNS enzyme activity in the blood, we strongly suspected a diagnosis of MPS IVA disease. To definitively diagnose, we measured GALNS enzyme activity in dermal fibroblasts; this assay showed a clinically significant low level (0.01 nmol/hour/mg protein) compared with normal levels (7.48–9.52 nmol/hour/mg protein). This finding established a definitive diagnosis of MPS IVA [9], also known as Morquio A syndrome.

### Treatment

At 30 months of age, treatment with elosulfase alfa enzyme replacement therapy (ERT) at 2 mg/kg/week was initiated and continued throughout the reported follow-up period (36 months after the start of treatment). Treatment did not begin before this date because ERT in Japan had not been approved. Both acetaminophen (10 mg/kg) and loratadine (5 mg) were given 30 minutes prior to ERT administration to reduce pain, fever, and allergic reaction.

### Outcome and follow-up

For this report, the progress of the patient was followed for 36 months, during which time he was adherent to treatment and assessed yearly.

Urine KS levels at the start of ERT were high (71.05 µg/mg creatinine) compared with the reference range (1.75–6.81 µg/mg creatinine) and decreased over time: 69.5%, 36.8%, and 47.1% of the pre-ERT level at 12 months, 24 months, and 36 months of treatment, respectively.

The patient grew 14.5 cm during the 36-month treatment period. To assess spinal deformity, a thoracolumbar MRI after 24 months of treatment showed deformity and hypoplasia of the vertebral body, hypoplasia of the odontoid process, and spinal canal stenosis at C2–C4 and Th2–Th4 (which worsened at 36 months). However, no neurological abnormalities were observed.

To assess physical endurance and exercise performance, a 6-minute walk test and a 3-minute stair climb test were conducted. The initial 6-minute walk test was conducted 12 months after the start of treatment; at 36 months the walking distance had increased by 68 m. The 3-minute stair climb test was conducted 24 months after the start of treatment; at 36 months the number of steps taken had decreased by 31.

## Discussion and conclusions

Given the rarity of Morquio A syndrome, and the complex and heterogeneous symptoms, diagnosis can be very challenging. Here we report the case of an early diagnosis in an infant boy within 2 months of initial presentation, based on the link with pectus carinatum bone deformity. This case exemplifies the importance of considering MPS disorders as part of the initial differential diagnosis of pediatric patients with skeletal deformities. Both urine GAG levels and a blood enzyme MPS panel are simple in-hospital or outsourced screening tests that could trigger early definitive diagnosis of Morquio A syndrome.

According to the International Morquio A Registry, the most common symptoms that trigger definitive diagnosis are short stature, genu valgum, kyphosis, and pectus carinatum [3, 10]. In our reported case the main symptom that triggered definitive diagnosis was pectus carinatum, which is typical of the disease. However, the Registry study found that, regardless of the presentation of these common symptoms, the lapse from presentation to diagnosis can still average almost 3 years [3]. There are likely two main reasons for this delay in diagnosis: the rarity of the disease and the complicated nature of a definitive diagnosis.

The prevalence of Morquio A syndrome has been reported to range from 1 in 76,000 births in Northern Ireland [11] to 1 in 667,000 in Japan [7]. This means that, with an annual frequency of only several cases, the disease and its typical symptoms would not be well known to non-specialist clinicians. In addition, pectus carinatum, one of the most common symptoms for diagnosis, can also be representative of a congenital thorax deformity, which has a frequency of 1 in 1500–2000 births [12]. As pectus carinatum is a typical finding of thoracic deformities and is generally not associated with other comorbidities, pediatric patients are often initially assigned to orthopedists rather than pediatricians [12], which could further hinder definitive diagnosis. Given that pectus carinatum could also be included in a differential diagnosis for osteodysplasia, Marfan’s syndrome, Poland’s syndrome, and the MPS diseases, we encourage all specialists to consider these options during clinical examination.

As mentioned above, and although initially described almost 100 years ago in 1929 [13], diagnosis of Morquio A syndrome can still be very challenging [9]. For example, agreement of clinical, radiographic, and laboratory findings is required, and radiographic images of many body regions are recommended. In addition, urine GAG levels may not be reflective of enzyme activity; additional enzyme activity analysis may be needed to exclude MPS IVB (which presents phenotypically similar [though milder] to Morquio A, but with a mutation in the β-galactosidase gene [9]), multiple sulfatase deficiency and mucolipidoses types II/III; and GALNS activity should be confirmed in leucocytes or cultured dermal fibroblasts. Further, even with this battery of tests, genetic analysis is recommended and may possibly be needed for confirmation [9]. A major issue is that non-specialist clinicians may not suspect Morquio A syndrome in their differential diagnosis; they will therefore not request, or may not be aware of, this battery of tests (including the availability of a commercial blood enzyme screening test) required for diagnosis, thereby delaying diagnosis until the appropriate specialist becomes involved in the case.

Early diagnosis of Morquio A may be critical to patient well-being, as it can lead to early treatment and therefore offer the potential for improved quality of life and survival [2]. Although ERT may not be able to halt the progression of all organ dysfunction, it may delay symptom progression or possibly prevent complications; for example, ERT may prevent cardiopulmonary dysfunction [14]. To obtain the best outcome for the patient, early identification of organ dysfunction followed by regular, multidisciplinary, comprehensive, and organized care by a team of specialists is required. In the case reported here, ERT began at 30 months of age, giving the patient a chance for improved quality of life. To ensure that early diagnosis of Morquio A is possible, we advocate for greater awareness of the mucopolysaccharidoses among the medical community.

## Data Availability

The data analyzed during the current study are not publicly available for privacy reasons.

## Abbreviations

C6S: Chondroitin-6-sulfate
ERT: Enzyme replacement therapy
GAGs: Glycosaminoglycans
GALNS: *N*-Acetylgalactosamine-6-sulfatase
KS: Keratan sulfate
MPS: Mucopolysaccharidosis
